# Management of pituitary adenoma with mass effect in pregnancy: a case report

**DOI:** 10.4076/1757-1626-2-6350

**Published:** 2009-09-08

**Authors:** UV Okafor, IO Onwuekwe, HU Ezegwui

**Affiliations:** 1 Department of Anaesthesia, University Of Nigeria Teaching Hospital (Unth), Enugu, Nigeria; 2 Department of Internal Medicine, University Of Nigeria Teaching Hospital, Ituku Ozalla, Enugu, Nigeria; 3 Department of Obstetrics and Gynaecology, University of Nigeria teaching hospital, Ituku Ozalla, Enugu, Nigeria

## Abstract

A middle aged primigravida was managed at the University of Nigeria Teaching Hospital, Enugu, Nigeria for a pituitary macroadenoma. She was admitted at 33 weeks gestational age following a history of blurred vision and generalized headache, worse on bending down. After neurological consultation and investigations, a diagnosis of pituitary macroadenoma with mass effect was entertained. A plan for neurosurgery after delivery was made and the patient put on bromocriptine to reduce tumour size. Premature labour at 35 weeks resulted in caesarean delivery of a live baby. She was managed in the intensive care unit for three days where oral bromocriptine was resumed before she was transfered to the postnatal ward. Within ten hours of the transfer, she developed accelerated hypertension with encephalopathy and had a cardiac arrest shortly afterwards. This rare case highlights both the possible role of bromocriptine as a cause of postpartum hypertension and the possible development of a sudden catastrophic intramoural infarction or hemorrhage (pituitary apoplexy) in a patient with a macroadenoma.

## Introduction

The preoperative management of patients with pituitary tumors presenting for surgery requires careful preoperative assessment and meticulous pre and postoperative management especially so in the pregnant patient. Particular problems in these parturient have to do with the primary hormonal hyper secretion and its complications, and to the mass effect of the tumours [1].

Knowledge of the normal physiology and anatomy of the pituitary gland is important to allow an understanding of path physiological effects relevant to anesthesia and of the potential complications that might arise perioperatively [1].

Anaesthesia for pituitary disease is a highly specialized area and because the reported cases are few [2], especially during pregnancy, extensive experience in this area is often lacking. Prolactinomas are the commonest pituitary tumors complicating pregnancy. Based on their size prolactinomas are classified into macroadenoma (>1 cm) or micro adenoma (<1 cm). In this case, we report the preoperative management of a parturient with a macroadenoma presenting for caesarean delivery at the gestational age of 34 weeks and five days.

## Case presentation

A 30 year old nulliparous Nigerian woman of the Igbo tribe presented for antenatal care at 24 weeks gestational age. She had two visits to the hospital before she presented to the hospital as an emergency. After a review by the obstetrician, she was admitted to the antenatal ward and a consult sent to the neurologist for a review.

The patient gave a two year history of blurring of vision, followed a month later by generalized headache that was worse on bending. The headache had intensified since becoming pregnant. Of recent there had been associated vomiting. There was also a history of menstrual irregularities, urinary frequency and protrusion of the right eye. She did not report any weakness in the limbs or trauma to the head or eye. She had visited an ophthalmologist where she was treated on outpatient basis.

Physical examination revealed a young woman in painful distress, conscious and well oriented. She was not pale, icteric or febrile to touch. Blood pressure on admission was 120/80 mmHg and pulse rate was 76 beats per minute, regular and of good volume.

Examination of the central nervous system revealed proptosis of the left eye and a blind right eye. There was temporal visual loss with reduced acuity in the left eye. Fundoscopy showed established optic atrophy in the right eye with early optic atrophy changes in the left eye. Other cranial nerves were intact with no sign of meningeal irritation. There were no asymmetries in the limbs. The possibility of an intracranial space occupying lesion was entertained.

Relevant investigations were ordered. A computerized tomography (CT) scan of the brain showed a large pituitary tumour (>10 mm) with pressure effects, occluding the anterior horn of the left lateral ventricle. The other ventricles were dilated.

The results of other investigations were as follows - serum prolactin 250 ng/ml (6.0-24), serum T3 2.4 ng/ml (0.6-1.6), serum cortisol 310 ng/ml (50-230) and fasting blood sugar 69 mg/dl (65-110). Abdominopelivic ultrasound scan revealed a live singleton fetus at 33 weeks with cephalic presentation and anterior placenta. The fetal heart rate was 130/minute.

A diagnosis of pituitary macroadenoma with mass effect was made.

The symptoms gradually regressed with good clinical improvement. After sixteen day of admission, the patient went into premature labor at 34 weeks and five days. After a review by the obstetricians, she was booked for emergency caesarean section and the anesthetists were informed.

Because the CT scan showed evidence of raised intracranial pressure, general anesthesia with the relaxant technique was used with sodium thiopentone, non-depolarizing muscle relaxant, opioid analgesics and oxygen/air. Sodium thiopentone was used for maintenance of anesthesia in intermittent bolus doses as that would prevent further rise in intracranial pressure and the possible risk of bleeding if the available volatile agent, halothane was used. Anesthesia by the corresponding author was uneventful and the patient recovered satisfactorily. She was taken to the intensive care unit for postoperative care and close monitoring.

She recovered full consciousness in ICU. On the second postoperative day, she was resumed on oral bromocriptine with the following postoperative drugs; ampiclox, metronidazole, pethidine and promethazine. The following day, she had complained of restlessness before she was discharged to the ward on the third postoperative day by 2 pm in fairly satisfactory condition. Later that day at about 11:30 pm, the duty doctors were called to see the patient on account of sudden and sustained rise in blood pressure. On examination she was unconscious and afebrile. The blood pressure was 210/110 mmHg and the pulse rate was 150 beats per minute.

A diagnosis of accelerated hypertension with encephalopathy was made and the following drugs prescribed; intravenous bolus dose of hydralazine 5 mg stat, then 40 mg in a litre of 5% dextrose in water to run at ten drops per minute with quarter hourly monitoring of blood pressure. About 15 minutes later the patient started gasping and went into cardiac arrest. Attempts at resuscitation failed. An autopsy was not done.

## Discussion

Prolactin secreting adenomas are the most commonly encountered pituitary tumours in women of child bearing age [3]. Because it interferes with the hypothalamic - pituitary - ovarian axis at various levels, it is believed to be responsible for about a third of all cases of female infertility.

The relationship between pregnancy and the growth of prolactinomas was first identified in the 1970s when it was observed that in women who had recovered fertility following treatment with ovulation inducing drugs or bromocriptine, pregnancy was associated with pituitary growth [4]. Some of these women later developed visual defects that required surgery while others had their vision restored to normal spontaneously after delivery [1].

Molitch has summarized the recent findings on the effects of pregnancies on prolactinomas [5]. While the risk of clinically significant enlargement for women with microprolactinomas is 1.3%, the risk of enlargement for women with untreated macroprolactinomas is 23.2%. There is a less likely chance of symptomatic growth during pregnancy after shrinkage with bromocriptine even after it is discontinue [5].

It has been suggested that re-introduction of bromocriptine is the preferred treatment for the pregnant woman with prolactinoma who becomes symptomatic. It has been reported that most cases quickly show regression of symptoms such as headache and signs (visual field changes) of tumour enlargement.

The history in this case appears inadequate as there is no indication of a possible use of bromocriptine before pregnancy as there was a positive history of menstrual irregularities.

There was clinical improvement in our patient after introduction of bromocriptine up to the time she went into labor at nearly 35 weeks. An emergency caesarian section was carried out under general anesthesia with the delivery of a live baby with an Apgar score of 8 after 5 minutes.

She was transferred to the intensive care unit for close monitoring and observation. She had a bout of pyrexia on the second postoperative day with a return to normal temperature after administration of an antipyretic. The patient had been screened for malarial parasites and treated for malaria before caesarean delivery. Other events were uneventful except for a complaint of restlessness, before she was discharged to the ward. Within the next ten hours, she had developed accelerated hypertension and died. Bromocriptine shares with other ergot alkaloids the property of vasoconstriction, with the potential for serious tissue injury [6]. Hypertension [7] and cardiac injury has been associated with bromocriptine use in the postpartum period [8].

Because of these rare but serious side effects which may also include cerebral angiopathy, strokes and seizures, its use is not advocated in the postpartum period [9]. Perhaps a lower starting dose of bromocriptine should have been used.

It was for this reason that oxytocin was used for uterine contraction following caesarean delivery in order to avoid the use of egometrine, another ergot alkaloid.

Though an autopsy was not carried out in this patient, a catastrophic complication of a pituitary tumour (especially a macroadenoma) related to sudden intratumoural infarction or haemorrhage (pituitary apoplexy) [10] might be a possibility.

## Conclusion

Prolactinomas are reportedly rare during pregnancy and are known to respond to treatment with bromocriptine. If the pregnancy is far from term and the clinical features are unresponsive to bromocriptine, surgery is advised. If near term, the woman is delivered before neurosurgery. Anesthesia for caesarean delivery and ICU care were fairly uneventful. The use of dopamine agonists like bromocriptine in the postpartum period should be reviewed. This rare case also highlights the pitfalls that clinicians may encounter in the management of such patients especially when related literature from the region is scarce.

## Competing interests

The authors declare that they have no competing interests.

## Consent

The patient is deceased and informed consent was obtained from the husband for publication of this case report and accompanying images. A copy of the written consent will be available for review by the Editor-in-Chief of this journal. Unfortunately he misplaced the CT-scan of the patient.

## Authors' contributions

All the authors contributed significantly, as there was a multidisciplinary case. The editing work was done mainly by UVO and IO. All the authors were in agreement on the final version of the manuscript.
